# Adeno-Associated Viral Gene Delivery of Wild-Type Human Tau Induces Progressive Hyperphosphorylation and Neuronal Cell Death in the Hippocampi of Middle-Aged Rats

**DOI:** 10.3390/cells14161238

**Published:** 2025-08-11

**Authors:** Ryan C. Gorzek, Aurelie Joly-Amado, Natalia Hurst-Calle, Graham L. Gabrielson, Maxine Miller, Sue Osting, Kevin R. Nash, Corinna Burger

**Affiliations:** 1 Department of Neurology, University of Wisconsin School of Medicine and Public Health, Madison, WI 53726, USA; 2 College of Letters and Science, University of Wisconsin-Madison, Madison, WI 53706, USA; 3 Department of Molecular Pharmacology and Physiology, University of South Florida, Tampa, FL 33620, USA; ajoly@usf.edu (A.J.-A.); nhurstcalle@usf.edu (N.H.-C.); nash@usf.edu (K.R.N.)

**Keywords:** tau, Alzheimer’s disease, adeno-associated virus, neurodegeneration, hippocampus, hyperphosphorylation, GFP

## Abstract

Tau aggregation and the subsequent formation of neurofibrillary tangles are hallmarks of Alzheimer’s disease (AD) and other dementias. While accumulation of tau aggregates is believed to contribute to cell death and neurodegeneration, tau aggregation and hyperphosphorylation are also correlated with cognitive impairment in AD. To understand the role of tau in neurodegeneration, we used adeno-associated virus serotype 9 (AAV9) to express human wild-type 4-repeat, 0-N-terminus tau isoform (AAV-htau) in the Cornu ammonis area 1 (CA1) region of the dorsal hippocampus of adult 6-month-old Fischer 344 rats. AAV expressing green fluorescent protein (AAV-GFP) or uninjected rats were used as controls. To characterize early phenotypes, we investigated pathological changes at 3, 8, and 12 weeks post-injection of AAV-htau. Our results show that at 3 weeks post-injection, there was already robust expression of human tau in the CA1 region of animals injected with AAV-htau compared to those injected with AAV-GFP or the uninjected controls. At 12 weeks post-injection, area CA1 showed a statistically significant reduction in cell number and a thinner neuronal layer all throughout the anterior dorsal hippocampus, as well as redistribution to the somatodendritic areas of CA1. We also found hyperphosphorylation of tau at all three timepoints. In spite of this pathology, we did not find any hippocampal-dependent cognitive impairment in rats overexpressing human tau. These results provide evidence of AAV-htau as a progressive model of tauopathy pathology to study changes in phosphorylation status and neuronal cell death that might precede cognitive impairment.

## 1. Introduction

Tauopathies, such as Alzheimer’s disease (AD), frontotemporal dementia with parkinsonism, and progressive supranuclear palsy, are an increasingly prevalent class of neurodegenerative diseases characterized, in part, by hyperphosphorylation and aggregation of the microtubule-associated binding protein tau [1,2]. However, our understanding of these diseases has been hindered by the variety of clinical neurogenerative presentations being evaluated post-mortem. To this day, the biochemical and neuropathological mechanisms of these diseases have yet to be explained, delaying attempts at the development of effective therapeutics.

Many earlier studies have evaluated tau aggregation in AD progression to better understand its role in the characteristic features of neurodegenerative diseases. In normal physiology, tau protein is widely distributed in the central nervous system and is predominantly located in the axons, where it aids in the dimerization and subsequent polymerization of tubulin through promotion of microtubule assembly and stabilization [3,4]. In AD, abnormal levels of phosphorylation cause tau to dissociate from the microtubules and aggregate into dimers, oligomers, and long twisted pairs of helical filaments, known as neurofibrillary tangles (NFTs) [5,6,7]. The accumulation of beta-amyloid plaques and tau aggregates are believed to contribute to cell death and neurodegeneration by interfering with synaptic transmission [8]. While tau aggregation and the formation of NFTs undoubtedly play a role in these diseases, the exact mechanisms leading to neuronal and synaptic loss and disease progression have not been fully described. More recently, a role for oligomers and not NFTs in neuronal dysfunction has been demonstrated in animal models [9,10,11]. It is of interest to us that tau pathology has been associated with cognitive impairment, which is the most devastating AD phenotype [12].

Transgenic rodent models of tauopathy have been widely used, relying on expression of murine or human isoforms in brain areas that are related to AD and other tauopathies [9,13,14,15,16]. Viral gene delivery models of tauopathy have also been described [17,18,19,20,21]. Although mouse models have greatly helped research, rat models offer several advantages over mouse models especially when it comes to neurodegenerative diseases. Indeed, rats have larger brains, which facilitates more precise surgical manipulations and better spatial resolution in imaging techniques, such as fMRI and PET. Additionally, rats exhibit more complex behaviors and cognitive functions, such as intricate social interactions [22], better performance in cognitive tests like the Morris water maze [23], and distinct patterns of impulsive and addictive behaviors [24], making them superior for behavioral studies. Physiologically and relevant to AD and other tauopathies, rats, like humans, have six isoforms of the tau protein [25]. These factors contribute to more robust and translatable findings in rat models, particularly in the context of neurodegenerative diseases, like Alzheimer’s disease. However, there is a limited number of existing transgenic rat models for AD [26] and only the TgF344-AD model develops tau pathology [27]. Thus, the purpose of this study was to test the feasibility of inducing tauopathy in non-transgenic rats through viral gene delivery and evaluate potential cognitive deficits in these animals.

In this study, we evaluated the early time course of pathological changes associated with the expression of full-length human wild-type 4-repeat, 0-N-terminus tau isoform (tau4R) in the rat hippocampus utilizing AAV9 and evaluated tau-mediated neurodegeneration, including early- and late-stage phosphorylation species, and how these tau pathology changes might affect cognitive impairment associated with AD. We selected 4R tau because, although both 3R and 4R tau are expressed in roughly equal amounts in the adult human brain [28], the use of 4R tau in the context of AAV expression remains understudied. 4R tau is particularly relevant in studies of microtubule dynamics, neuronal transport, and cytoskeletal integrity, as it binds microtubules more tightly and stabilizes them more effectively than 3R tau [29]. This biochemical property makes 4R tau especially important for maintaining cytoskeletal integrity and axonal transport in mature neurons. Additionally, 4R tau is more prone to aggregation in certain tauopathies, like progressive supranuclear palsy, corticobasal degeneration, argyrophilic grain disease, and globular glial tauopathy, whereas 3R is more relevant in Picks disease [28]. Finally, the 4R tau used in this study is a non-mutant form, making it more representative of tauopathies that occur without tau gene mutations. Our study illustrates that while this model might be useful to assess specific aspects of tau pathology, it also highlights the broader utility of this rapid and efficient method for generating somatic transgenic rats. This approach enables the investigation of tau pathology progression and provides a valuable platform for elucidating the molecular mechanisms underlying tau hyperphosphorylation, toxic oligomer formation, preceding cognitive impairment.

## 2. Methods

### 2.1. Animal Subjects

Subjects were 6-month-old male Fischer 344 rats purchased from Envigo. Rats were given ad libitum access to water and Teklad 14% protein rodent pellets (2014, Envigo, Indianapolis, IN, USA) and maintained on a 12 h dark/light cycle for 3-, 8-, or 12- weeks. All animal procedures were approved by the University of Wisconsin Institutional Animal Care and Use Committee and conducted in accordance with the United States National Institutes of Health Guide for the Care and Use of Laboratory Animals.

### 2.2. Adeno-Associated Viral Vectors and Stereotactic Injections

Adeno-associated viral vector (AAV) preparations of serotype 9 were purified as previously described [30]. Viral transduction with human tau (AAV-htau) was achieved using vectors carrying the intron-free, human wild-type 4-repeat, 0-N-terminus *tau* sequence under control of the hybrid cytomegalovirus enhancer/chicken β-actin (CBA) promoter. Viral preparations were produced following methods described in detail by us [30]. Control transduction with green fluorescent protein (AAV-GFP) was achieved using vectors carrying the UF11 sequence [31], which contains the humanized *GFP* sequence under control of the CBA promoter. UF11 was purchased from the University of Florida vector core. The viral vector doses used were 3.9 × 10^10^ vector genomes per hemisphere for AAV-tau and 1.4 × 10^10^ vector genomes per hemisphere for AAV-GFP. Bilateral intracerebral injection of AAV vectors into the dorsal rat hippocampus to target areas Cornu Amonis areas 1 and 3 (CA1-CA3) has been previously described in detail by us [32]. Two sites in each hemisphere were infused with 2 μL of viral preparation per injection site to cover the entire rostral-caudal dorsal hippocampus. Following surgery, rats were given at least 2 weeks to recover in their home cages prior to biochemical and immunohistochemical analyses.

### 2.3. Behavioral Assays

All behavioral assays were conducted during the dark cycle and in the following order: Morris water maze, followed by contextual and cued fear conditioning. Two weeks following surgery and prior to the Morris water maze, the rats were handled by the experimenters for 4 days. The experimenters were blinded to the treatment of the animals throughout behavioral testing and analysis.

#### 2.3.1. Morris Water Maze 

Rats were tested in the 2-day version of the Morris water maze [33]. The task consisted of 1 day of visible platform training (4 trials), followed by 1 day of hidden platform training (4 trials) and a probe trial. A group of animals was tested 2–3 weeks post-injection, and a different group was tested at 12 weeks post-injection. Both proximal and distal visual cues were present. The animals were recorded by a ceiling mounted camera using VideoTrack v3.22 (ViewPoint Life Sciences Inc., Montreal, QC, Canada).

#### 2.3.2. Fear Conditioning

Contextual and cued fear conditioning were conducted using a Startle and Fear Combined System (Panlab, Harvard Apparatus, Holliston, MA, USA). Using load cells beneath the chamber floor (load cell amplifier gain set at 2000), this system monitored animal activity throughout the duration of fear conditioning sessions.

On training day, the rats were placed in a black rectangular chamber, with a clear plastic door and grid floor, inside a sound attenuation box. The 7 min training session began with 2 min of silent exploration, followed by a 30 s tone (2000 Hz, 80 dB). During the last 2 s of the tone, a mild footshock (0.8 mA) was delivered. Subsequently, the session contained another 2 min of silent exploration and a 30 s tone, terminating in a footshock. The training session ended with a 2 min period of silent exploration, following which the rats were returned to their home cage. The rats were returned to the identical chamber 24 h following the training session to assess contextual fear memory recall. The contextual test session was 7 min of silent exploration. Approximately 1 h after the contextual test (25 h after the training session), cued fear memory recall was assessed. The rats were placed in a distinct chamber inside the sound attenuation box, with a white circular interior, smooth floor, and the scent of vanilla. The 7 min cued test session was identical to the training session, without footshocks delivered at the end of tone presentations. For contextual and cued fear conditioning, comparisons were made between the groups using the percentage of time spent freezing over the entire 7 min free exploration testing protocol. Additionally, the percentage of time spent freezing during 30 s increments for contextual fear conditioning testing were compared to determine whether learning deficits were a result of early extinction. Differences in average freezing time during training, both prior to the introduction of sound or shock stimuli and in the two-minute exploration period following the first sound/shock combination, were compared to rule out any mobility issues as a result of treatment influencing the testing results.

#### 2.3.3. Behavior Data Analysis

Morris water maze. The time to find the platform was recorded for each trial of visible and hidden platform training. For the probe trial, time spent in the platform quadrant and the number of entries into the platform zone were recorded.

Fear conditioning. PACKWIN 2.0 software (Panlab, Barcelona, Spain) was used to convert recordings from chamber load cells into animal activity levels, based on a set of experimenter-defined parameters. Animal activity was defined as freezing behavior based on a set of parameters (Threshold Up = 100 (arbitrary units), Threshold Down = 14, Minimum Freezing Duration = 2 s). The percentage of time spent freezing during the entire 7 min contextual test session was used to assess contextual fear memory. The average percentage of time spent freezing during the two 30-s tones in the cued test session was used to assess cued fear memory.

All statistical analyses were performed using Prism 10 (Graphpad Software Inc., La Jolla, CA, USA) and expressed as the mean ± standard error of the mean (SEM). Significance was set at *p* < 0.05. Two-way ANOVA with multiple comparisons was used to determine significance.

### 2.4. Crude Protein Extraction Procedure

At 3, 8, or 12 weeks post-injection, the animals were deeply anesthetized with isoflurane gas, their brains were immediately removed after decapitation, and the whole hippocampi were dissected, snap-frozen in liquid nitrogen, and stored at −80 °C until processing for crude extraction. Tissue was lysed using a Dounce homogenizer at a ratio of 200 µL/50 mg tissue in RIPA buffer [in mM: 150 NaCl, 10 Tris-HCl (pH 7.4), 1 EDTA; 1%Triton X-100, 0.1% SDS, 0.1% sodium deoxycholate], containing Protease Inhibitor Cocktail (1:100; Prod# P8340, Sigma-Aldrich, St. Louis, MO, USA) and Phosphatase Inhibitor Cocktails 2 and 3 (1:100; P5726 (Cocktail 2) and Prod# P0044 (Cocktail 3), Sigma-Aldrich). Homogenized hippocampal solutions were centrifuged at 15,000× *g* for 20 min at 4 °C, after which supernatants were collected and their protein concentrations were determined by bicinchoninic acid assay (Pierce^TM^ BCA Protein Assay Kit, Cat# 23225, Thermo Fisher Scientific, Waltham, MA, USA).

### 2.5. Western Blotting

First, 15 μg of crude extract protein in Laemmli sample buffer was heated for 10 min at 70 °C, separated on 4–15% gradient SDS–PAGE gels (Mini-PROTEAN TGX, Bio-Rad, Hercules, CA, USA) and transferred to PVDF membranes (Trans-Blot Turbo Transfer Pack; Prod # 1704156, Bio-Rad) using a Trans-Blot Turbo Transfer System (Bio-Rad). Following transfer, the membranes were incubated in Revert^TM^ 700 Total Protein Stain for Western Blot Normalization (LI-COR Lincon, NE, USA catalog # 926-11011). Primary antibodies included human tau (HT7; 1:2000; MN1000, Invitrogen, Thermo Fisher Scientific), rat-specific tau (1:1000; #829601, BioLegend, San Diego, CA, USA), pThr 231(1:5000; #701056, Invitrogen, Thermo Fischer Scientific), and pSer396 (1:4000, #44752G, Invitrogen, Thermo Fischer Scientific), AT8 (1:2000, #MN1020, Invitrogen, Thermo Fisher Scientific). Membranes were blocked with Tris-buffered saline-Tween20 and 5% milk fat (HT7, Tau5, Rat Tau), or Intercept^TM^ blocking buffer [LI-COR #927-60001 (pThr231, AT8, pS396)] for one hour at room temperature, prior to overnight primary antibody incubation at 4 °C. The primary antibodies were incubated in a 2.5% solution of their respective blocking agent. Following washes, the membranes were incubated in IRDye 680RD or 800CW secondary antibodies (1:10,000; 925-32213, 925-32212, 925-68073, 925-68072, LI-COR, Lincoln, NE, USA) before imaging with the LI-COR CLx Imaging System.

#### Western Blot Statistical Analyses

Quantification and analysis of protein bands were performed using Empiria Studio Software v2.3 (LI-COR) https://www.licor.com/bio/help/empiria_studio/index.html (accessed on 18 May 2025). Immuno-positive bands were normalized to a total amount of protein detected by Revert 700^TM^, and analyses were performed following the manufacturer’s protocol (see Supplementary Figures S1 and S2 for Revert images of the protein gels and full images of the Western blots shown in the results section). Statistical analyses were performed using Prism 10 (GraphPad Software Inc., La Jolla, CA, USA). One-way-ANOVA, followed by Tukey’s multiple comparison tests, and 2-way ANOVA with multiple comparisons were employed for statistical analysis of Western blot data.

### 2.6. Histology

#### 2.6.1. Tissue Processing

At 3, 8, or 12 weeks following vector injection, the animals were deeply anesthetized with isoflurane gas. Perfusion and tissue processing were conducted as previously described in detail [34]. Brains were sectioned in the coronal plane at a 45 μm thickness for immunohistochemical analysis.

#### 2.6.2. Tau Immunohistochemistry

All solutions were prepared with a buffer consisting of PBS with 2% bovine serum albumin (BSA; Calbiochem, La Jolla, CA, USA) and 0.1% saponin (Sigma-Aldrich). The sections were washed, blocked in buffer with 20% normal serum for 45 min, incubated overnight in primary antiserum (HT7 MN1000: 1:1000; pSer202/Thr205 phosphorylated tau [AT8] MN1020, Invitrogen, Thermo Fisher Scientific: 1:2000) in 1% normal serum. After washes in PBS + BSA + saponin, the sections were incubated in biotinylated secondary IgG (1:300; Vector, Burlingame, CA, USA; Cat #: BA-1000) for 3 h, followed by 1 h in avidin-biotin complex (Standard Elite kit; Vector; RRID: AB_2336819). Final visualization was carried out with 0.04% 3,3′–diaminobenzidine (DAB; from tablets; Sigma-Aldrich) and 0.01% H_2_O_2_ in PBS, pH 7.4.

#### 2.6.3. Nissl Staining

Sections were mounted on subbed slides, dehydrated using graded concentrations of ethanol, cleared with histoclear, rehydrated, soaked in cresyl violet stain, dehydrated once again using graded concentrations of ethanol, cleared with histoclear, and coverslipped with Eukitt (Cat # 50-192-8500; Fisher Scientific, Hampton, NH, USA). Slides were scanned on a Zeiss Axioscan slide scanner (Zeiss, White Plains, NY, USA) and analyzed with NearCyte image analysis software v2.1.3 (http://www.nearcyte.org), as previously published. Data are shown as the percentage area positively stained, which we have previously shown correlates to neuron number [35].

#### 2.6.4. Histological Image Acquisition

Light microscopic images were obtained with a digital camera (Q Imaging Retiga 2000R; Nikon Instruments, Melville, NY, USA) on a Nikon E600W Eclipse epifluorescent microscope (Nikon Instruments). Brightfield images were acquired at an initial 36-bit to 1 scale and saved as 16-bit files. Images were prepared for reproduction in Photoshop 2025 (Adobe, Mountain View, CA, USA). Adjustments on scale, gamma, contrast, and hue and subsequent sharpening with the unsharp mask algorithm were applied to the entire image. Montages were assembled with no subsequent retouching. Digital images of each slide and its corresponding sections were analyzed using custom image analysis software (Nearcyte, Zeiss) to quantify the pixel-positive area fraction based on defined thresholds. For each rat, values from all sections were averaged to yield a single representative value per brain region for statistical analysis. All hippocampal images were acquired by an investigator blinded to the experimental conditions (N = 8 sections per animal).

#### 2.6.5. Gallyas Staining

Gallyas staining was performed according to previously established methods [36]. Prior to staining, tissue sections were mounted on pre-coated slides. The slides were then treated with 5% periodic acid for 5 min, rinsed with water, and sequentially incubated in silver iodide for 1 min and 0.5% acetic acid for 10 min. They were subsequently placed in a developer solution containing 2.5% sodium carbonate, 0.1% ammonium nitrate, 0.1% silver nitrate, 1% tungstosilicic acid, and 0.7% formaldehyde. To stop the reaction, the slides were treated with 0.5% acetic acid, followed by incubation in 0.1% gold chloride and immersion in 1% sodium thiosulfate. After a final rinse in water, the slides were dehydrated and coverslipped. Data are shown as the percentage area positively stained.

## 3. Results

### 3.1. Gene Delivery of Human Tau Results in Robust Human Tau and Phosphorylated Human Tau Expression Throughout Hippocampal Areas CA1/2

**Figure 1:**
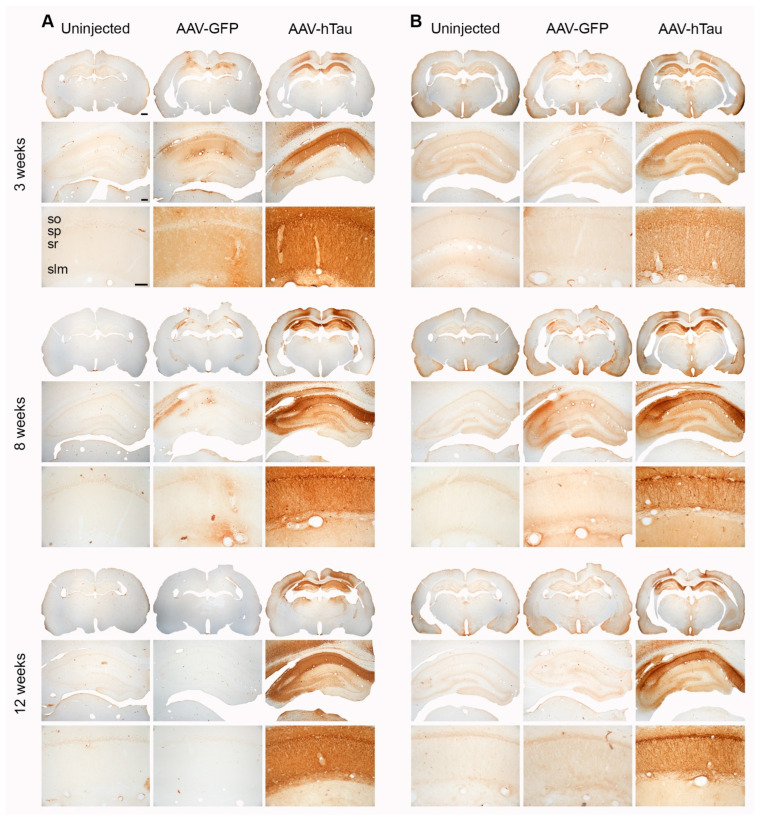
Human tau immunohistochemistry shows robust transgene expression in AAV-htau-injected hippocampi. (**A**) Representative sections showing human tau (HT7 antibody) immunoreactivity at the three timepoints, at three different magnifications. (**B**) Representative sections showing pSer202/Thr205 (AT8 antibody) immunoreactivity at the different timepoints at three different magnifications. Stratum pyramidale (SP), stratum oriens (SO), stratum radiatum (SR), and stratum lacunosum-moleculare (SLM); N = 4 brains per group/timepoint. Scale bars: 1× = 1 mm, 4× = 250 µm, and 20× = 100 µm.

To assess the effects of human tau overexpression, we injected the dorsal hippocampi of 6-month-old male Fischer 344 rats with AAV9 vectors carrying the wild-type tau 4R sequence (AAV-htau). Control rats were injected with AAV expressing the humanized GFP sequence (AAV-GFP) or were left uninjected. We qualitatively assessed the distribution of total human tau in the dorsal hippocampus at 3, 8, and 12 weeks post-injection using a primary antibody that recognizes human tau (HT7) using immunohistochemical analysis (Figure 1A). In the AAV-htau injected rats, robust HT7 immunostaining was observed throughout stratum pyramidale (SP), stratum oriens (SO), stratum radiatum (SR), and stratum lacunosum-moleculare (SLM) of hippocampal areas CA1 and CA2, while overlying cortical areas and CA3 appeared only partially transduced. As expected, no significant specific stain was found in uninjected animals, although there was some non-specific staining in some AAV-GFP injected animals at 3 weeks post-injection that persisted at 8 weeks but not 12 weeks, which could be due to residual inflammation at the site of injection [37].

Next, we assessed the distribution of human tau phosphorylated at Ser202/Thr205 (AT8, paired helical filament human tau, PHF-tau) [7,38] (Figure 1B). AT8 immunostaining was similar in distribution to HT7 3 weeks post-injection of AAV-htau, with overt staining throughout SP, SO, SR, and SLM of hippocampal areas CA1 and CA2. Overlying cortical areas and CA3 also appeared only partially transduced. At 8 weeks post-injection, pSer202/Thr205 immunostaining was markedly increased and densely labeled in pyramidal cell bodies and their proximal dendrites within SR in AAV-htau injected animals (Figure 1B). Less frequently, we also observed pSer202/Thr205 (AT8) immunoreactivity concentrated in pyramidal dendrites in SO and more distally within SR. In AAV-tau rats, this dense somatodendritic staining pattern observed 8 weeks post-injection was also apparent 12 weeks post-injection (Figure 1B). Starting at 8 weeks post-injection, we observed some overtly less dense regions in area CA1 that may be indicative of major neuron loss, which persisted until 12 weeks. pSer202/Thr205 immunostaining was not observed in the uninjected animals, but we occasionally saw faint staining in AAV-GFP-injected hippocampi (Figure 1B), which could be due to residual inflammation at the site of injection. We did not see any fibrillary aggregates by Gallyas staining at any of the time points (Figure S3).

### 3.2. Gene Delivery of Human Tau Increases Total Hippocampal Tau Levels at 3, 8, and 12 Weeks Post-Injection and Results in an Increase in Phosphorylated Tau

**Figure 2:**
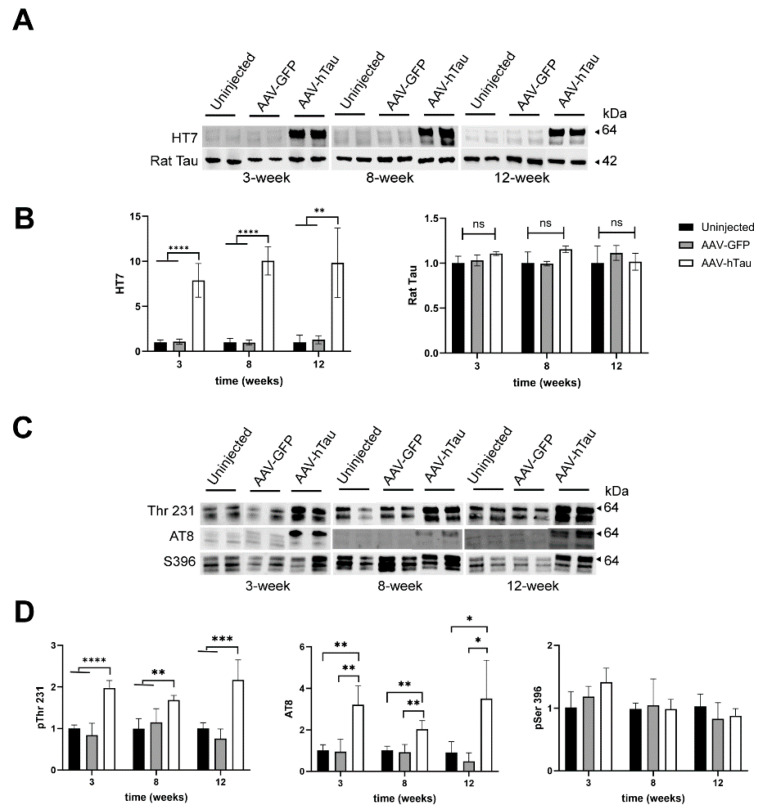
Timeline of expression of tau and phosphorylated tau in the hippocampus of rats. (**A**) Representative Western blots showing immunoreactive bands for human and rat tau for the different treatments and timepoints. (**B**) Bar graph showing the quantification of each immunoreactive band. A total of N = 4 replicates per treatment per time point were used for statistical analysis. Since HT7 only recognized human tau, HT7 expression levels are presented as normalized levels relative to total protein. Rat tau levels of expression were calculated as ratios relative to uninjected levels of expression. (**C**) Representative Western blots showing immunoreactive bands for phosphorylation of tau at different amino acid residues at different treatments and timepoints. (**D**) Quantification of expression of different phosphorylated residues. pThr 231, pSer202/Thr205 (AT8), and pSer396 expression for the different conditions were calculated as ratios relative to uninjected levels of expression. *= *p* < 0.05, **= *p* < 0.01, ***= *p* < 0.001, ****= *p* < 0.0001, ns = not significant.

Next, we quantified human tau protein levels by Western blot analysis of whole-hippocampus crude protein preparations. HT7 reactivity (normalized to total amount of protein detected by Revert 700; see Methods Section for details and Figure S1A,B) was significantly enhanced in AAV-htau rats at 3, 8, and 12 weeks post-injection, relative to uninjected or AAV-GFP-injected animals (Figure 2A,B) (one-way ANOVA, main effect of treatment, F_(2,9)_ = 51.5, *p* < 0.0001 [3 weeks]; F_(2,9)_ = 120.8, *p* < 0.0001 [8 weeks]; F_(2,9)_ = 19.14 *p* = 0.0006 [12 weeks]). We also quantified the expression of a rat-specific isoform of tau (42 kDa) and found that levels of this isoform were not significantly changed with treatment (Figure 2A,B). Two-way mixed ANOVA with multiple comparisons revealed no statistical effect of time for HT7 or rat tau.

To evaluate whether the level of phosphorylated tau was altered in rats expressing human tau, total levels of human tau phosphorylated at Thr231 [(pThr231) early-stage tau aggregation and hyperphosphorylation marker that precedes formation of oligomers)] [6,38], tau phosphorylated at Ser202/Thr205 (AT8 antibody and hyperphosphorylation marker that precedes pre-tangle formation) [39], and at Ser396 [(pSer396) late-stage tau aggregation] [7,38] were examined (for images of the full blots, see Figure S2A–C). We found the main human tau species was the hyperphosphorylated full-length form (64 kDa) at all time points [17,40,41]. A significant increase in tau phosphorylation at Thr231 was observed in the AAV-tau rats beginning at 3 weeks, which was sustained at 8 and 12 weeks post-injection, relative to the controls (Figure 2C,D; one-way ANOVA, main effect of treatment, F_(2,9)_ = 37.7, *p* < 0.0001 [3 weeks]; F_(2,9)_ = 8.80, *p* = 0.0076 [8 weeks] F_(2,9)_ = 22.06, *p* = 0.0003 [12 weeks]). Tau phosphorylation at pSer202/Thr205 (AT8) was also significantly increased at 3, 8, and 12 weeks post-injection, relative to the controls (Figure 2C,D; one-way ANOVA, main effect of treatment, F_(2,9)_ = 16.26, *p* = 0.001 [3 weeks]; F_(2,9)_ = 12.99, *p* = 0.0022 [8 weeks] F_(2,9)_ = 8.39, *p* = 0.0088 [12 weeks]). There was no statistically significant difference in the phosphorylation levels between the uninjected and AAV-GFP groups in any of the timepoints examined. pSer396 reactivity in the AAV-htau rats was not significantly different than that in the controls at any time point (Figure 2C,D). Two-way mixed ANOVA with multiple comparisons revealed no statistical effect of time for pThr231, pSer202/Thr205, or pSer396.

### 3.3. Degeneration of CA1 Pyramidal Neurons Is Observed Following Gene Delivery of Human Tau

**Figure 3:**
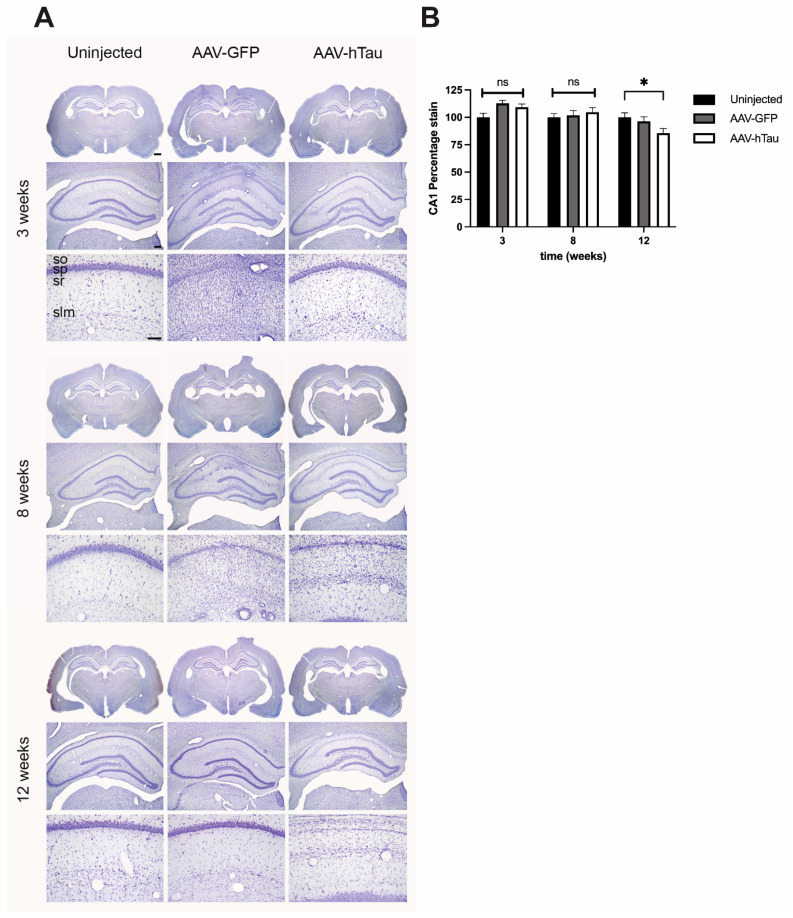
Nissl staining of hippocampal sections showed progressive neuronal loss in AAV-htau-injected hippocampi. (**A**) At 3 weeks post-injection, all experimental groups showed normal hippocampal anatomy (N = 4 rats per group/timepoint). At 8 weeks, thinning of layer CA1 was noticeable in AAV-htau-injected animals when compared to uninjected. Some AAV-GFP injected animals also displayed thinning of CA1. Neurodegeneration of CA1 was significant at 12 weeks post-injection when compared to uninjected or AAV-GFP-injected hippocampi. (**B**) Densitometry analysis showed that at 12 weeks, area CA1 of AAV-htau-injected hippocampi had significant cell loss relative to controls, * *p* = 0.042. ns= not significant. Scale bars: 1× = 1 mm, 4× = 250 µm, and 20× = 100 µm.

To assess the neurotoxicity of AAV-htau delivery, we conducted Nissl staining to quantitatively assess cellular architecture and neuron loss within the dorsal hippocampus. At 3 weeks post-injection, we observed no differences in stratum pyramidale morphology between AAV-htau and AAV-GFP or uninjected rats (Figure 3 top panel). At both 8 and 12 weeks post-injection in AAV-htau, SP in area CA1 contained noticeably fewer cell bodies than age-matched controls and AAV-htau rats at 3 weeks post-injection (Figure 3 middle and lower panels). Pyramidal neuron loss appeared restricted to area CA1; little morphological difference was observed between the AAV-tau rats and uninjected controls in areas CA2 and CA3 (Figure 3). Quantification of Nissl staining by densitometry analysis showed that there was significant cell loss only at 12 weeks post-injection in the AAV-htau-injected hippocampi versus the controls (ANOVA, F_(2,135)_ = 3.255, *p* = 0.042).

### 3.4. Overexpression of Human Tau Does Not Result in Hippocampal-Dependent Learning Impairment

**Figure 4:**
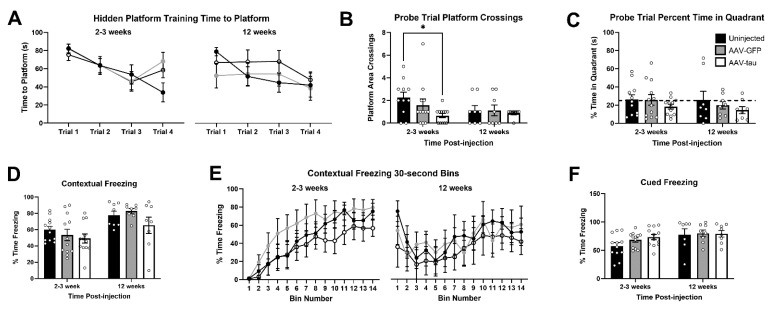
AAV-htau-injected animals did not display cognitive impairment at the 2–3- or 12-week time points. (**A**) Latency to reach the platform during the four trials of hidden platform testing (2–3 weeks: N = 12 animals for each group (uninjected, AAV-GFP, and AAV-htau; 12 weeks: N = 8 for each group). (**B**) AAV-htau rats swam in the area across the former location of the hidden platform significantly less than the uninjected rats at the 2–3 week time point. *= *p* = 0.015. (**C**) All experimental groups spent similar amounts of time swimming in the target quadrant at both timepoints. (**D**) No differences were found in the percentage of freezing time during contextual testing 24 h post-training. (**E**) Freezing was evaluated every 30 s for the 7 min silent exploration period. No differences in extinction were found at either point between the experimental groups. (**F**) All experimental groups were able to associate the auditory cue.

Human tauopathies are characterized by cognitive impairment. To determine the effect of tau pathology on hippocampal-dependent learning and memory behavior of AAV-htau-injected rats, animals were tested in the Morris water maze and fear conditioning behavioral tasks at 2–3- and 12-week time points. At 2–3 and 12 weeks post-injection, we observed no differences in the latency to find the hidden platform between the AAV-htau and AAV-GFP or uninjected rats (Figure 4A). There was a statistically significant difference between the uninjected and AAV-htau groups in probe trial platform crossings only at the 2–3-week time point (Figure 4B, two-way ANOVA with Tukey’s multiple comparisons test; F_(2,53)_ = 2.34, *p* = 0.015). No differences were found in the percentage of time spent in the hidden platform quadrant (Figure 4C). The AAV-htau rats did not exhibit deficits in the contextual fear condition paradigm when compared to the AAV-GFP or uninjected rats at 2–3 or 12 weeks post-injection. There was no difference in their ability to associate the context 24 h post-training at either timepoint (Figure 4D). Additionally, the percentage of time spent freezing during 30 s increments for context testing were compared to determine whether learning deficits were a result of early extinction (Figure 4E). All animals had similar levels of freezing during the 7 min of silent exploration. No statistically significant differences were found in the percentage of freezing to the auditory cue (Figure 4F).

## 4. Discussion

In this study, we evaluated the early time course of pathological and cognitive changes associated with the expression of human tau4R in the rat hippocampus, as a potential model to study mechanisms underlying cognitive impairment and pathology associated with tauopathies. Our data show that AAV-htau was successful at rapidly increasing the levels of human tau in the rat hippocampal CA1 region, which is susceptible to aging and neurodegeneration [42]. Indeed, it reached steady levels of expression at 3 weeks post injection, which could be sustained up to 12 weeks post-injection (Figure 1A and Figure 2A,B). At this early time point, we also found robust expression phosphorylated pSer202/Thr205 (AT8; Figure 1B and Figure 2C,D). This was accompanied by increased levels of phosphorylated tau at Thr231, which correlates with pThr231 increase in patients in early stages of the disease [38]. Similarly, levels of phosphorylation of pSer202/Thr205, which appear to occur at pre-tangle formation stages [39], were significantly higher in animals overexpressing human tau relative to the controls at the three time points (Figure 2C,D). No differences were found in the levels of phosphorylation of the late-stage hyperphosphorylation marker pSer396.

We first detected statistically significant hippocampal neuronal loss at 12 weeks post-injection (Figure 3A,B) namely in areas CA1/CA2 (Figure 3A). Similar results were observed by Jaworski et al., 2009 [17]. In this study, they observed a more rapid neuronal death progression. Namely, rapid neuronal loss in area CA2 was evident as early as 1.5 weeks post-injection, followed by loss of CA1 starting at 3 weeks with total neuronal loss at 12 weeks post-injection. This study used different viral vector doses and demonstrated that a high dose of 10^8^ transduction units produced the most severe phenotype. Jaworski et al. and other studies have also used the P301L mutation, which may have a more severe phenotype than wild-type human tau [17,43,44].

We also found immunostaining for HT7 and AT8 in some of the AAV-GFP-injected animals, but Western blot quantification showed no statistically significant levels of hyperphosphorylation of tau in the AAV-GFP-injected hippocampi (Figure 2). Similarly, neuronal loss was apparent in the Nissl-stained AAV-GFP hippocampi, and although it was not statistically significant (Figure 3A,B), this was probably due to the AAV9-GFP-associated immune reaction [45]. Expression of AAV9-GFP but no other serotypes resulted in a reduction of dopamine in the striatum in animals injected in the substantia nigra (SN), although no cell loss was found in SN [46]. The use of AAV-GFP as a control has been controversial, and researchers have proposed other controls to account for AAV-dependent transgene transcription and translation. As an example, Jaworski et al. used a tau isoform lacking the microtubule binding domain as a negative control [17]. However, in another study by this group, the use of AAV-GFP did not result in dendritic degeneration when compared to the effects of AAV-tauP301L [18]. The use of GFP has recently also been suggested to cause some toxic effects with long-term expression [37]. It is still important to use a gene-coding vector as a negative control to account for viral-encoded mRNA-mediated quenching of cellular translational preinitiation complexes.

Our data show gradual neuronal loss in the hippocampal region and an increase in tau hyperphosphorylation and mislocalization of tau to the somatodendritic area of CA1. We see neurodegeneration without NFT formation, as assessed by Gallyas staining (Figure S3), which is in agreement with the report by D’Orange et al. who observed that expression of AAV-htau only resulted in NFT formation when co-expressed with a pro-aggregation tau peptide [20]. Similarly, Jaworski et al. found degeneration without the formation of NFTs in mice injected either with wild-type or P301L AAV-htau [17]. On the other hand, Cook et al. demonstrated NFTs when injecting AAV-htau P301L in neonate mice and evaluated tau pathology at six months of age. The differences could be due to the use of the mutant form of tau and time of expression [19].

Surprisingly, we did not find any cognitive impairment in AAV-htau rats, considering the thinning of area CA1 and hyperphosphorylation in that region. A report by Dayton et al. found similar results showing lack of memory impairment using wild-type AAV-htau when memory for the platform location in the Morris water maze was tested 12 weeks post-injection [21]. Conversely, Cook et al. observed behavioral deficits in mice injected at P0 with AAV-P301L and tested at six months of age [19]. The observed differences could be attributed to the duration of transgene exposure, as tau accumulation needs to reach a certain threshold before early and late markers of tauopathies become evident, as well as viral vector dose.

However, the effects on tau hyperphosphorylation and neuronal loss on cognition might be influenced by the animals’ age, as age is a risk factor for neurodegeneration and cognitive aging. Therefore, future studies could consider injecting animals at an older age and for longer periods of time to determine if neurodegeneration manifests more rapidly and results in cognitive impairment [19,20]. In summary, this model provides an avenue to study some aspects of tauopathy, in particular the timeline of tau hyperphosphorylation resembling that in patients with AD that precedes cognitive impairment [12]. However, proper controls and testing of viral vector preparations to ensure consistent phenotypes are warranted.

## 5. Conclusions

In summary, our study demonstrates that AAV-mediated expression of human tau4R in the rat hippocampus induces early and sustained tau hyperphosphorylation, neuronal loss, and somatodendritic mislocalization of tau, particularly in the CA1 region. These pathological changes occur in the absence of neurofibrillary tangle formation and without detectable cognitive impairment within the 12-week timeframe, highlighting the importance of tau accumulation thresholds, transgene duration, and animal age in modeling tauopathy-related cognitive decline. Indeed, future studies should incorporate later age time points, as aging may play a critical role in the development of neurofibrillary tangles and the emergence of cognitive impairments, potentially enhancing the translational relevance of this model. While our findings align with previous reports using wild-type tau, they also pinpoint the need for careful selection of viral vector controls and experimental parameters. This model offers a valuable platform to investigate early tauopathy mechanisms and their progression, providing insights into the temporal dynamics of tau pathology that precede cognitive symptoms in Alzheimer’s disease and related disorders.

## Data Availability

The raw data supporting the conclusions of this article will be made available by the authors on request.

## Supplementary Materials

The following supporting information can be downloaded at: https://www.mdpi.com/article/10.3390/cells14161238/s1, Figure S1: Full blots for Figure 2A for the 3-, 8- and 12-week timepoints; Figure S2: Full blots for Figure 2C for the 3-, 8- and 12-week timepoints; Figure S3: Gallyas staining of coronal sections from the hippocampus and corpus callosum of AAV-htau, AAV-GFP and uninjected controls for the 3-, 8- and 12-week timepoints.
