# Supplementary material for: Adeno-Associated Viral Gene Delivery of Wild-Type Human Tau Induces Progressive Hyperphosphorylation and Neuronal Cell Death in the Hippocampi of Middle-Aged Rats
Source: Cells. 2025 Aug 11;14(16):1238. doi: 10.3390/cells14161238 (PMC12384227; doi:10.3390/cells14161238)

Supplemental Figures

Figure S1. Full blots for figure 2A for the 3-, 8- and 12-week timepoints. Revert staining of western blots showing total protein loaded and whole blot image delineating the area selected for quantification for HT7 (S1A) and Rat tau signal (S1B) shown in figure 2A for the 3-, 8- and 12-week timepoints. Notice that the samples were not ordered as in the final figure (= uninjected, GFP tau), so the images had to be cut and pasted in the final figure 2 to follow that order. All samples were run in the same gel. Delineated box represents area excluded from analysis. Biorad Precision Plus Protein™ Kaleidoscope™ Prestained Protein Standards were used for these experiments.

S1A.

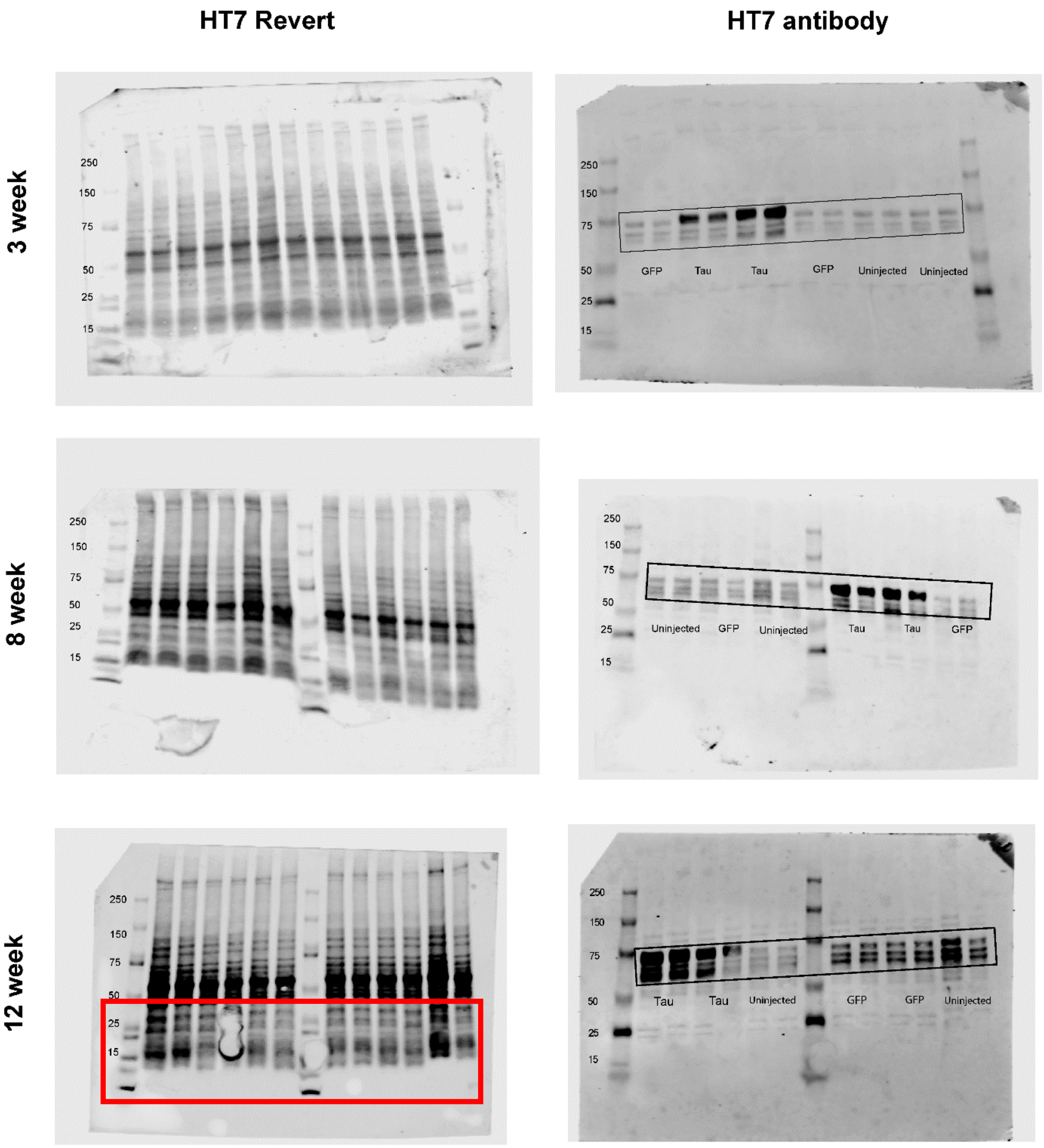

S1B.

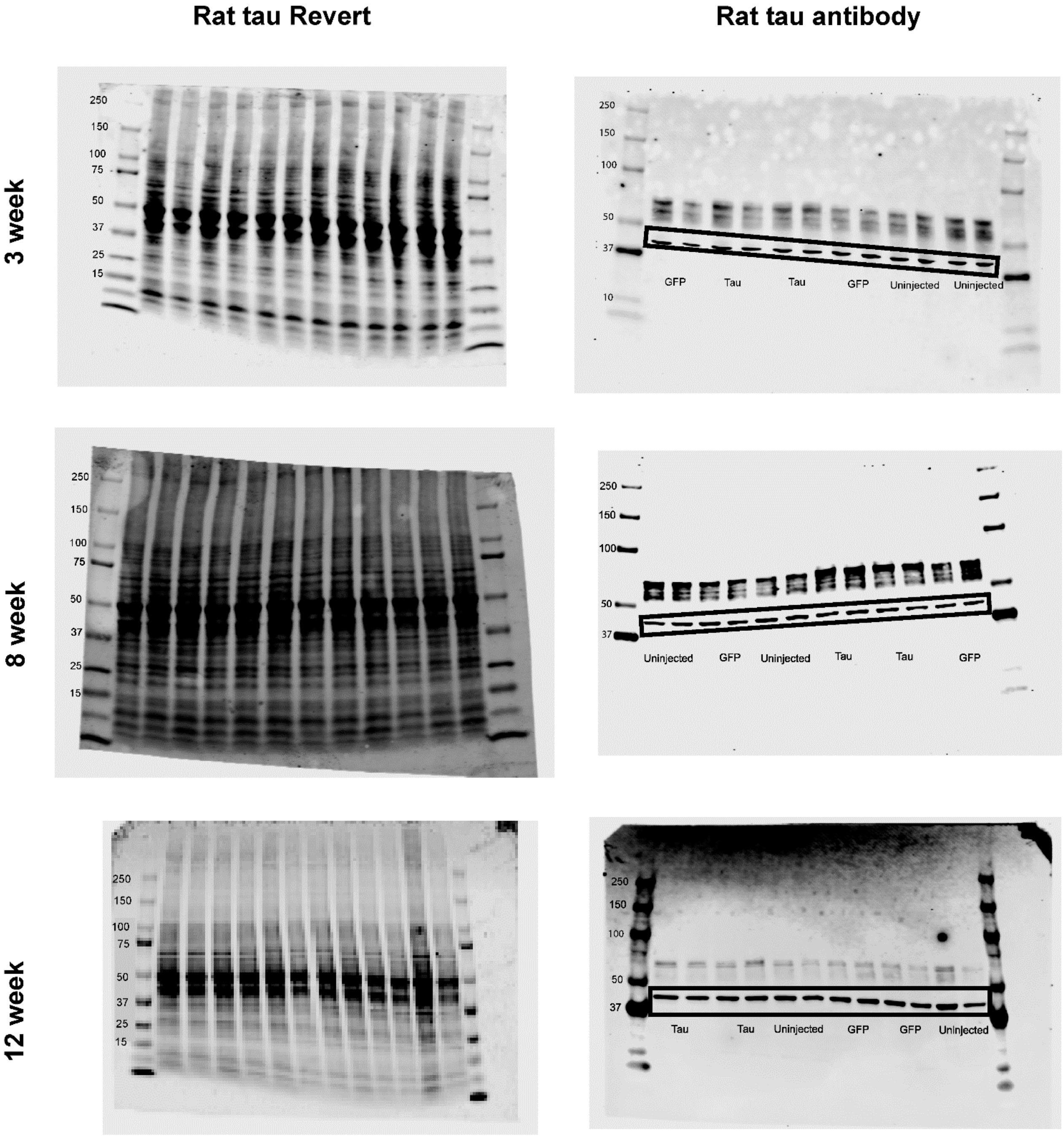

Rat tau Revert
Rat tau antibody
3 week
8 week
12 week
250
150
100
75
50
37
25
15
10
GFP
Tau
Tau
GFP
Uninjected
Uninjected
Uninjected
GFP
Uninjected
Tau
Tau
GFP
Tau
Tau
Uninjected
GFP
GFP
Uninjected

Figure S2. Full blots for figure 2C for the 3-, 8- and 12-week timepoints. Revert staining of western blots showing total protein loaded and whole blot image delineating the area selected for quantification for pThr231 (S2A) and pSer396 (S2B) and (S2C) pSer202/Thr205S (AT8) tau signal shown in figure 2C for the 3-, 8- and 12-week timepoints. Notice that the samples were not ordered as in the final figure (uninjected, GFP tau), so they had to be cut and pasted in the final figure to follow that order. All samples were run in the same gel.

S2A.

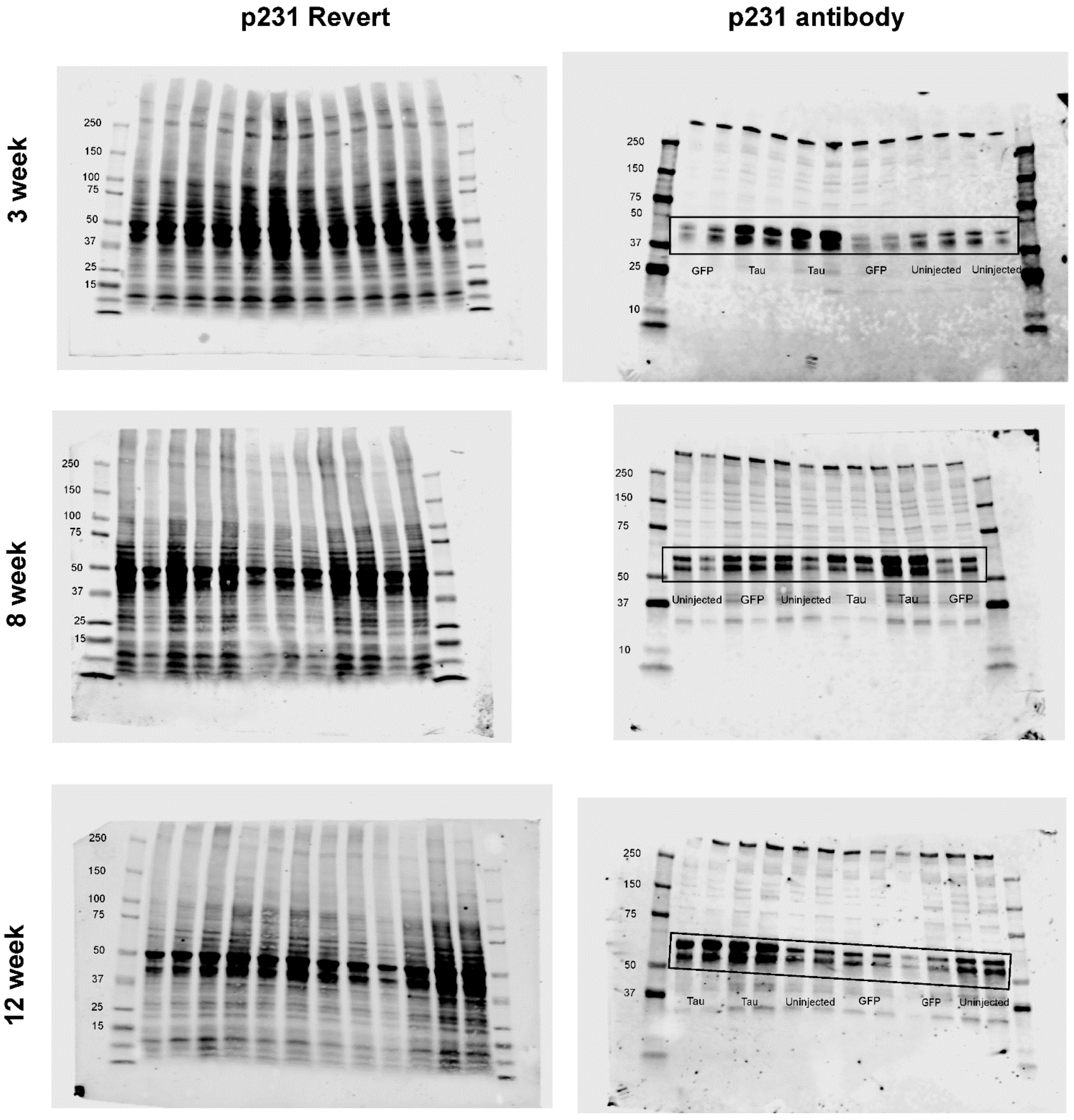

S2B.

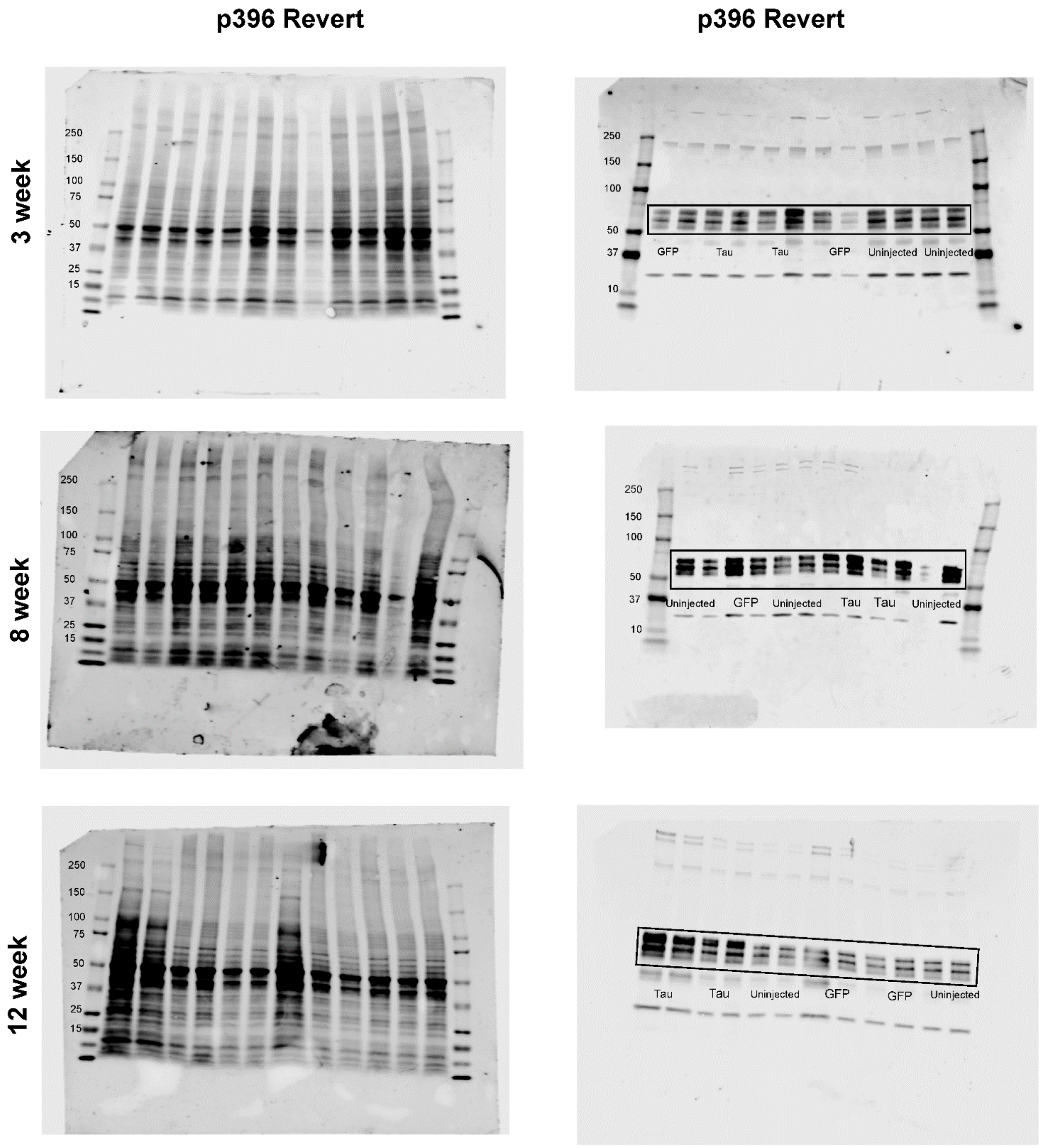
p396 Revert
p396 Revert
3 week
8 week
12 week
250
150
100
75
50
37
25
15
10
GFP Tau Tau GFP Uninjected Uninjected
Uninjected GFP Uninjected Tau Tau Uninjected
Tau Tau Uninjected GFP GFP Uninjected

S2C.

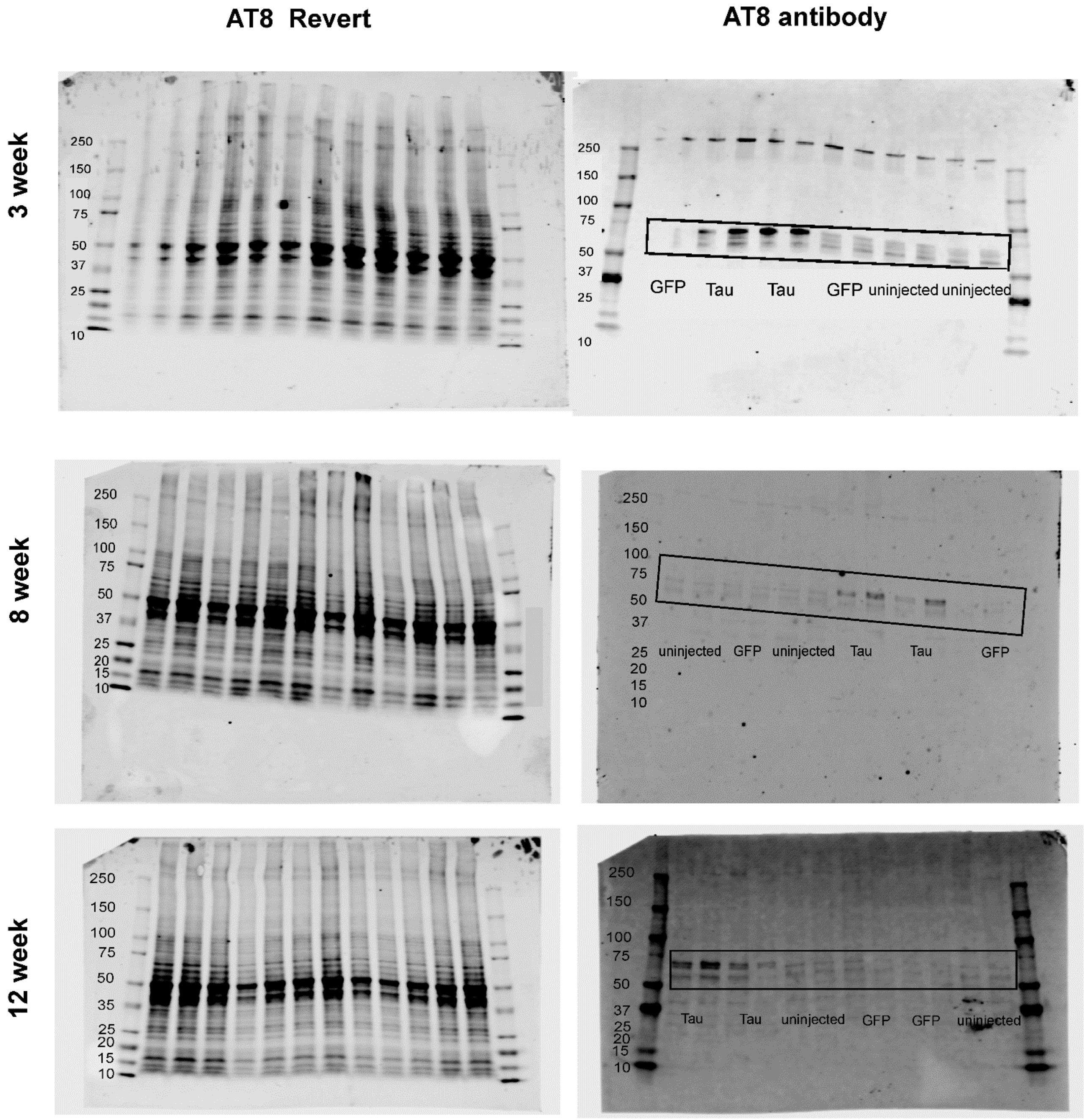
AT8 Revert
AT8 antibody
3 week
8 week
12 week
250
150
100
75
50
37
25
10
GFP
Tau
Tau
GFP
uninjected
uninjected
250
150
100
75
50
37
25
20
15
10
uninjected
GFP
uninjected
Tau
Tau
GFP
250
150
100
75
50
35
37
25
20
15
10
Tau
Tau
uninjected
GFP
GFP
uninjected

Figure S3. Top panels: Gallyas staining of coronal sections show no positive silver stained tau tangles in the hippocampus, regardless of age or treatment (n=4 rats per group/timepoint). Delineated box represents area included for analysis. Bottom panels: Positive signal was observed in the corpus callosum (cc) in all animals, consistent with Gallyas staining of myelin fibers. Scale bars top panels 1x = 1mm, bottom panels 5X = 200 µm.

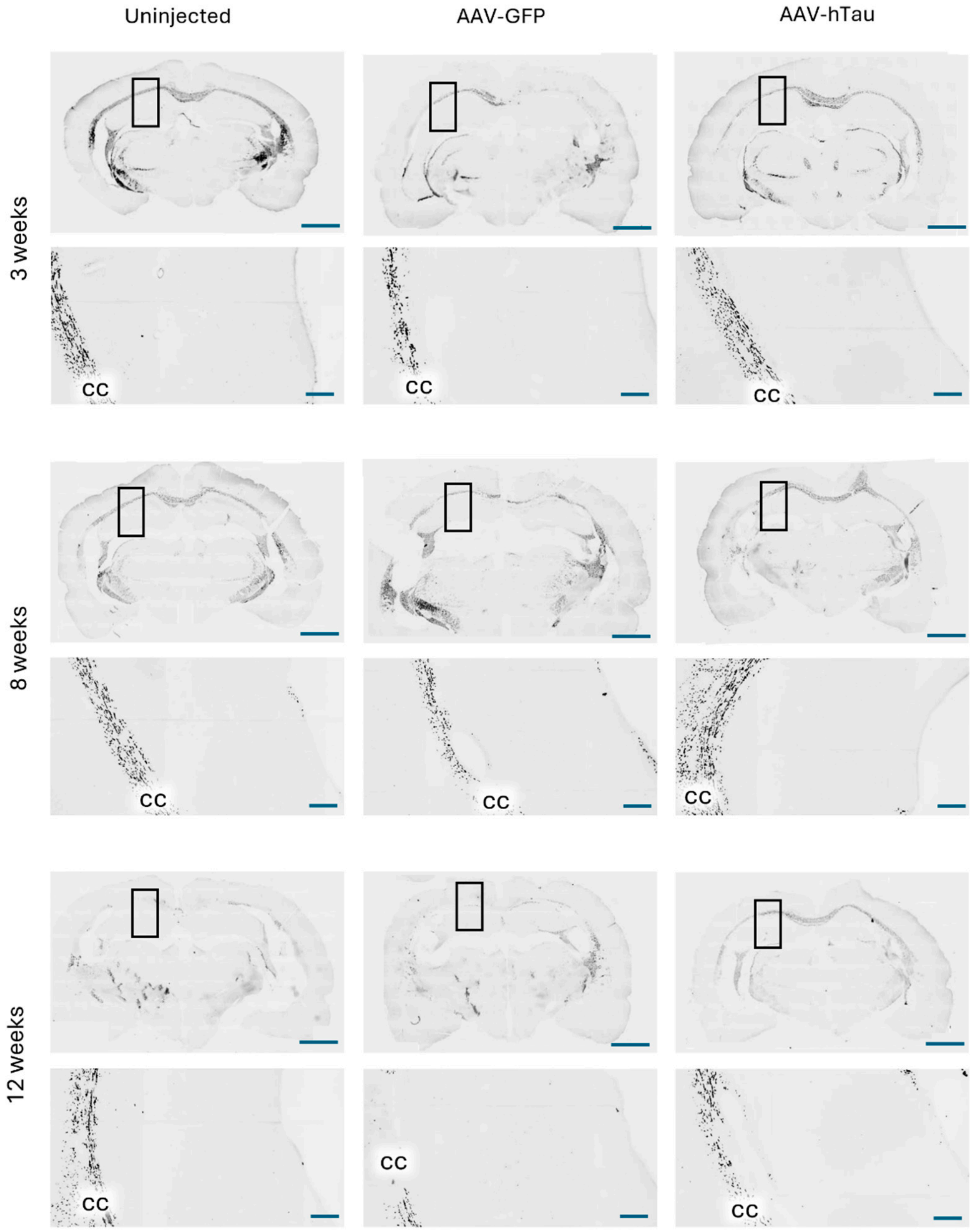

Supplement: Supplementary file 1 [file cells-14-01238-s001.zip › cells-3680673-supplementary.pdf]
